# 2cChIP-seq and 2cMeDIP-seq: The Carrier-Assisted Methods for Epigenomic Profiling of Small Cell Numbers or Single Cells

**DOI:** 10.3390/ijms232213984

**Published:** 2022-11-12

**Authors:** Congxia Hu, Jun Wu, Pengxiao Li, Yabin Zhang, Yonglin Peng, Ruiqi Liu, Wenfei Du, Yani Kang, Jielin Sun, Ji Wu, Zhifeng Shao, Xiaodong Zhao

**Affiliations:** 1 Key Laboratory of Systems Biomedicine (Ministry of Education), Shanghai Center for Systems Biomedicine, School of Biomedical Engineering, Shanghai Jiao Tong University, Shanghai 200240, China; 2 Center for Bioinformatics and Computational Biology, East China Normal University, Shanghai 200241, China; 3 Key Laboratory for the Genetics of Developmental & Neuropsychiatric Disorders (Ministry of Education), Bio-X Institutes, Shanghai Jiao Tong University, Shanghai 200240, China

**Keywords:** low-input ChIP-seq, low-input MeDIP-seq, single-cell ChIP-seq, female germline stem cells

## Abstract

Chromatin immunoprecipitation coupled with high-throughput sequencing (ChIP-seq) can profile genome-wide epigenetic marks associated with regulatory genomic elements. However, conventional ChIP-seq is challenging when examining limited numbers of cells. Here, we developed a new technique by supplementing carrier materials of both chemically modified mimics with epigenetic marks and dUTP-containing DNA fragments during conventional ChIP procedures (hereafter referred to as 2cChIP-seq), thus dramatically improving immunoprecipitation efficiency and reducing DNA loss of low-input ChIP-seq samples. Using this strategy, we generated high-quality epigenomic profiles of histone modifications or DNA methylation in 10–1000 cells. By introducing Tn5 transposase-assisted fragmentation, 2cChIP-seq reliably captured genomic regions with histone modification at the single-cell level in about 100 cells. Moreover, we characterized the methylome of 100 differentiated female germline stem cells (FGSCs) and observed a particular DNA methylation signature potentially involved in the differentiation of mouse germline stem cells. Hence, we provided a reliable and robust epigenomic profiling approach for small cell numbers and single cells.

## 1. Introduction

Understanding transcription dynamics during development requires investigation of *cis*-regulatory elements encoded in the genome. Chromatin immunoprecipitation (ChIP) coupled with deep sequencing (ChIP-seq) is a powerful method for characterizing the global epigenetic marks associated with *cis*-regulatory elements and the interaction between transcription regulators and genomic DNA [1,2]. However, the conventional ChIP-seq analysis requires a large number of cells (>10^6^ cells), thus impeding its application in rare cell populations [3,4].

The challenge of applying ChIP-seq to low sample inputs results from ChIP DNA loss during sample preparation procedures and the low efficiency of immunoprecipitation. In the past decade, many efforts have been made to enable low-input ChIP-seq analysis. Two pioneering studies demonstrated that a number of individual epigenetic marks could be detected in low-input ChIP samples [5,6]; and many efforts have been made to develop techniques allowing ChIP-seq analysis with limited cell numbers (<10^4^ cells), as summarized in a recent report [7]. These methods carried out in vitro transcription or linear amplification of low amounts of ChIP DNA (LinDA-seq [8] and TCL-ChIP [9]), and enabled the subsequent ligation-based ChIP library preparation. To increase immunoprecipitation efficiency, a microfluidic device was developed and introduced into the ChIP assay [10,11,12]; alternatively, ‘carrier chromatin’ (the exogenous chromatin with the same epigenomic modification) was implemented for immunoprecipitation (cChIP) [6,11]. By reducing the reaction volume, ULI-NChIP-seq [3,13] and STAR ChIP-seq [14] used micrococcal nuclease for chromatin fragmentation. Other methods adopted Tn5 transposase-mediated integration for library construction (ChIPmentation [15], ChIL-seq [16], Cut&Tag [17], itChIP-seq [18], CoBATCH [19], FACT-seq [20], and TIP-seq [21]) by incorporating several biochemical reactions in ChIP (e.g., the chromatin preparation, immunoprecipitation, and library construction) into one reaction, thus alleviating sample DNA loss. Some approaches utilized protein A- or antibody-conjugated micrococcal nuclease to generate fragmented chromatin DNA and characterize histone modifications or transcription factor (TF) occupancy in low-input samples [22] or single cells [23,24,25]. Although these methods have made rapid progress, they either require instruments or are less straightforward. Moreover, an epigenomic profiling strategy that allows the generation of epigenomic maps of either histone modifications or DNA methylation for low input is unattainable.

**Figure 1:**
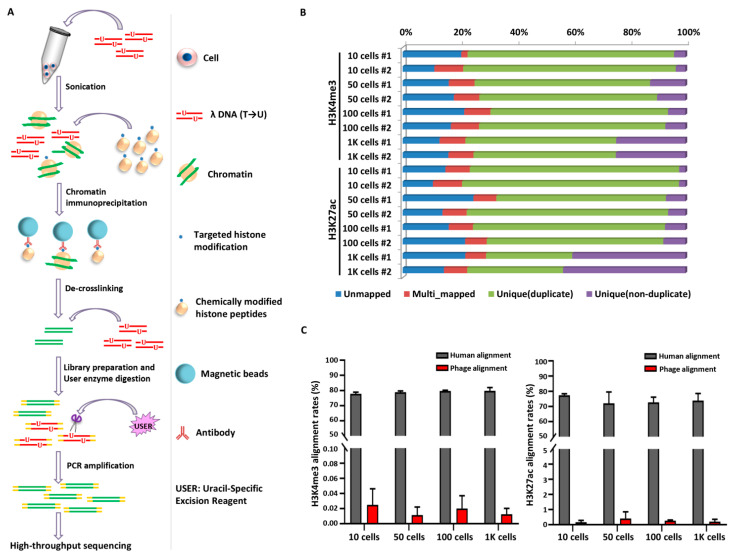
2cChIP-seq strategy overview and data quality presentation. (**A**) The scheme illustrates the conceptual design and key steps of the 2cChIP-seq protocol. The dUTP-containing lambda DNA fragments and chemically modified histone peptides are added as carriers during ChIP DNA preparation and library generation. (**B**) Deep sequencing features of 2cChIP-seq reads. (**C**) The plot depicts the proportions of 2cChIP-seq reads aligned to the human or lambda reference genome. Data were obtained from two independent experiments. The labels (#1 and #2) represent two 2cChIP-seq replicates.

Here, we envisioned a robust, straightforward, and microfluidic device-independent approach suitable for the epigenomic profiling of small samples and single cells. In this study, we adapted a carrier-assisted ChIP-seq (cChIP-seq) [26,27] approach by supplementing both chemically modified peptides and dUTP-incorporated DNA fragments for immunoprecipitation and library preparation; we named this protocol 2cChIP-seq (Figure 1A). Our improvement significantly increases immunoprecipitation efficiency and reduces DNA loss, which allows genome-wide epigenomic profiling of samples containing as few as 10 cells. In particular, 2cChIP-seq is basically compatible with conventional ChIP-seq procedures.

Although bisulfite sequencing could generate a single-base resolution methylome map, the high cost, limited signal recovery, and huge degradation of input DNA during bisulfite conversion make it less efficient for characterizing the methylome of a limited number of cells [28]. Therefore, we developed our carrier-assisted strategy to characterize methylome and demonstrated its efficiency by profiling histone marks or genomic DNA methylation in different types of cells. In this study, we provided an easy-to-use protocol for epigenomic analysis with a low sample input. Moreover, we extended this method to the single-cell level, measuring histone modification signals in hundreds of single cells simultaneously.

## 2. Results

### 2.1. Rational of 2cChIP-seq

2cChIP-seq introduces carrier materials, and basically, the procedures are performed as in conventional ChIP-seq, with the following exceptions: 1. the dUTP-containing lambda DNA fragments are supplemented during chromatin fragmentation and sequencing adaptor ligation; 2. the chemically-modified histone peptides are included for immunoprecipitation (Figure 1A). The supplementation of lambda DNA and modified peptides substantially reduces sample loss and facilitates immunoprecipitation and library preparation. Particularly, the spiked dUTP-containing lambda DNA could be removed from the resulting ChIP DNA library by uracil-specific excision reagent (USER) enzyme treatment. With this strategy, we generated epigenomic maps with low-cell-number samples.

### 2.2. 2cChIP-seq Efficiently Maps Histone Modifications with Low Sample Input

We firstly investigated the performance of 2cChIP-seq using K562 cell line chromatin equivalent to 10, 50, 100, and 1000 cells with the antibodies against H3K4me3 and H3K27ac, respectively. The 2cChIP-seq libraries with an average size of 400–500 bp DNA fragments were subjected to high-throughput sequencing (Figure S1). More than 75% of total qualified reads were mappable, and the deduplicated unique reads, ranging from 2% (10 cells)–43% (1000 cells), were used for downstream analysis (Figure 1B and Table S1). The ratios of read alignment to the lambda reference genome are no more than 0.040% (H3K4me3) and 0.725% (H3K27ac) (Figure 1C), indicating that the supplemented lambda DNA was almost removed from the 2cChIP-seq libraries.

**Figure 2:**
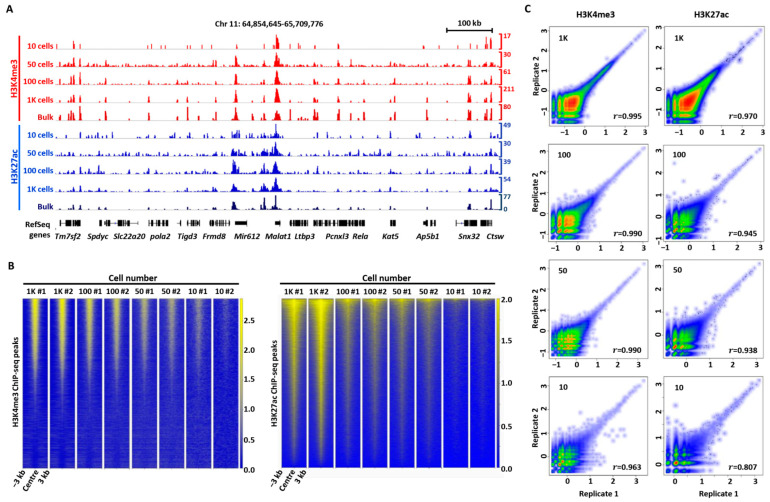
2cChIP-seq reliably generates H3K4me3 and H3K27ac profiles in small numbers of cells. (**A**) Normalized H3K4me3 and H3K27ac 2cChIP-seq signals at the indicated regions using data from various sample sizes. ENCODE data (H3K4me3, GSM2534289; H3K27ac, and GSM733656) are shown for comparison. (**B**) Heatmaps of 2cChIP-seq data for the indicated cell numbers. Data are centered on peaks called from GSM2534289 for H3K4me3 and GSM733656 for H3K27ac. A window of 6 kb (−3 kb to +3 kb) around the peak center is shown. (**C**) Scatter plot comparison of two 2cChIP-seq biological replicate datasets generated with various numbers of cells. The *r* indicates Pearson’s correlation coefficient calculated in non-overlapped 4-kb bins across the entire human genome. Sample size: *n* = 2. Chr, Chromosome. #1 and #2, represent two 2cChIP-seq replicates.

In particular, we observed that both H3K4me3 and H3K27ac peaks of bulk-cell ChIP-seq were well recapitulated by 2cChIP-seq (Figure 2A). With the locations of histone modifications from ENCODE datasets as benchmarks, we visualized 2cChIP-seq read density in a heatmap around 3 kb surrounding the locations of H3K4me3 or H3K27ac of ENCODE data, observing remarkable enrichment for all cell numbers examined (Figure 2B). Moreover, we acquired considerable signals of these histone marks in 10 cells, as demonstrated by the heatmap at ±3 kb of the transcription start sites (TSSs) of all genes (Figure S2A). To evaluate the reproducibility of the 2cChIP-seq method, we examined Pearson’s correlation coefficients of various sample sizes and observed high correlations between two biological replicates of each sample size: 0.807–0.963 for 10 cells, 0.938–0.990 for 50 cells, 0.945–0.990 for 100 cells, and 0.970–0.995 for 1000 cells (Figure 2C and Figure S2B). The values of FRiP were about 21–38% for 1000 cells and 13–17% for 100 cells (Figure S2C), being substantially higher than the 1% guideline of ENCODE data [29]. These results evidence that ChIP DNA was efficiently enriched and the 2cChIP-seq data were highly reliable.

### 2.3. Comparison of 2cChIP-seq with Other Reported Epigenomic Profiling Methods for Low Sample Input

**Figure 3:**
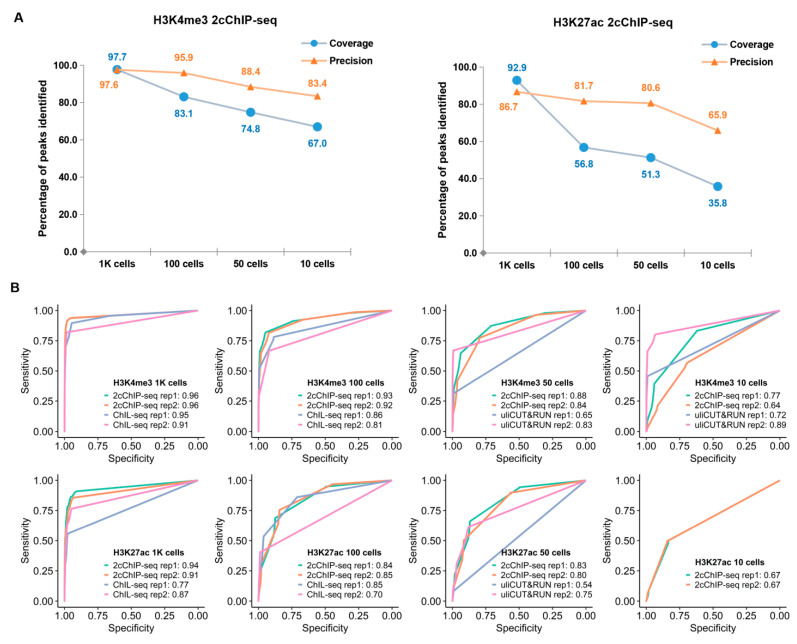
Performance comparison between 2cChIP-seq and other epigenomic profiling methods. (**A**) Line charts showing the coverage and precision of 2cChIP-seq peaks in the promoter region compared with those of ENCODE ChIP-seq data (H3K4me3, GSM2534289; H3K27ac, GSM733656). (**B**) ROC curves for the comparison of H3K4me3 and H3K27ac, respectively. ROC curves were constructed by comparing the data generated by distinct methods for low sample input with published data. The raw data of ChIL-seq and uliCUT&RUN are from GSE115047 and GSE111121, respectively. The values shown are for the area under the ROC curve (AUC).

We next compared the performance of 2cChIP-seq with other low-input epigenomic profiling methods reported recently. Compared to ENCODE H3K4me3 ChIP-seq data, 97.7% and 83.1% of H3K4me3 signals were recovered by 2cChIP-seq data of 1000 and 100 cells, respectively (Figure 3A). Moreover, 2cChIP-seq data exhibited 97.6% (1000 cells) and 95.9% (100 cells) precision rates (Figure 3A), performing better than those of H3K4me3 ChIL-seq [16]. A similar performance was observed in H3K27ac 2cChIP-seq data (Figure 3A).

Using the H3K4me3 and H3K27ac ChIP-seq data of ENCODE as the gold standard, we further performed receiver operating characteristic (ROC) curve analysis [30,31] to evaluate the data quality generated with 2cChIP-seq and other low-input epigenomic profiling methods (Figure 3B and Table S2). H3K4me3 2cChIP-seq exhibited better performance than those of uliCUT&RUN (10 and 50 cells) [23] and ChIL-seq (100 and 1000 cells) [16]. A similar result was also observed when the performance of distinct approaches for H3K27ac profiling was compared (Figure 3B).

### 2.4. Development of Single-Cell 2cChIP-seq for Epigenomic Profiling

**Figure 4:**
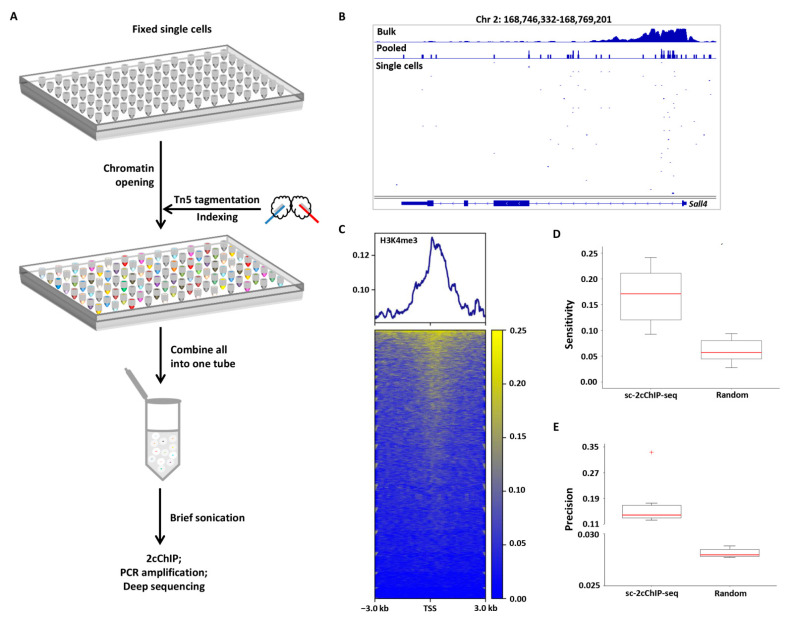
2cChIP-seq profiles of H3K4me3 signals in single cells. (**A**) Workflow of single-cell 2cChIP-seq. (**B**) Integrative Genomics Viewer (IGV) track view showing the H3K4me3 profiles from ENCODE bulk-cell ChIP-seq data (GSM1003756), pooled single-cell 2cChIP-seq data (from two independent experiments), and 12 individual cells. (**C**) Heatmap showing H3K4me3 signals at the TSS ±3 kb regions of genes for all single cells. (**D**,**E**) Boxplots showing the scores of sensitivity (**D**) and precision (**E**) for the top 5% of individual single cells, and simulated random genomic regions.

Considering that 2cChIP-seq allows the generation of reliable epigenomic profiles of less than 100 cells, we attempted to extend this approach to hundreds of single cells. For this purpose, we introduced Tn5 transposase-based indexing and tagmentation to 2cChIP-seq. As illustrated in Figure 4A and Figure S3, single cells were distributed into 96-well plates, followed by chromatin opening and tagmentation using Tn5 complexes with distinct combinations of T5 and T7 barcodes (Table S3). To improve sample recovery, dUTP-containing lambda DNA fragments were added to the tagmentation reaction as carrier. Single cells with distinct indexes were then pooled in one Eppendorf tube for immunoprecipitation with the low-input 2cChIP method. The purified ChIP DNA was ready for library amplification using i5 and i7 PCR index primers (Table S3), followed by deep sequencing (Figure S3B and Figure S4B).

Using this approach, we profiled H3K4me3 signals in 192 individual mouse (embryonic stem cells) ESCs (96 single cells per assay). About 6500 unique reads per cell were obtained (Table S4), much higher than those of Drop-ChIP [11] and scChIC-seq [24]. Compared with two alternative single-cell ChIP-seq reports [23,24], our method exhibited an advantage of a higher mapping rate (89% versus 1% in uliCUT&RUN, 6% in scChIC-seq). Additionally, the aggregated H3K4me3 signals from two batches of single-cell 2cChIP-seq experiments were highly correlated (*r* = 0.90) (Figure S4C). The pooled H3K4me3 reads of the single cells showed a pattern similar to those of H3K4me3 ChIP-seq data generated from bulk-cell sample (Figure 4B and Figure S4D) and were enriched around the TSSs (Figure 4C). We calculated the sensitivity and precision metrics of single-cell 2cChIP-seq, which yielded an average sensitivity of 0.17 and a precision of 0.14 (Figure 4D,E). These results suggest this technique is suitable for single-cell ChIP-seq analysis.

### 2.5. 2cMeDIP-seq Efficiently Profiles DNA Methylome in Low Numbers of Cells

**Figure 5:**
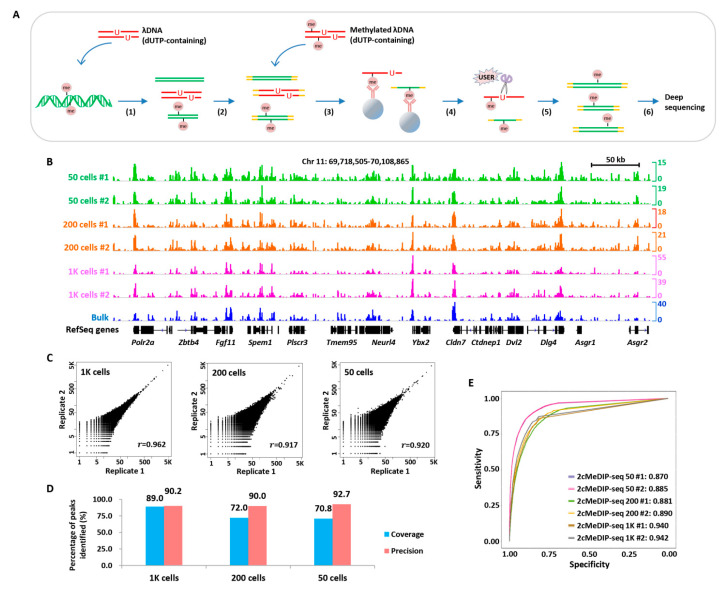
2cMeDIP-seq generates high-quality DNA methylone profiles with limited numbers of cells. (**A**) Overview of 2cMeDIP-seq protocol. Following (1) sample preparation and sonication, dUTP-containing λ DNA (lambda DNA) fragments are added as carrier, (2) repair of DNA ends and ligation of barcoded adaptors, (3) immunoprecipitation with antibody, methylated λ DNA (dUTP-containing) fragments are added as carrier, (4) reverse-crosslinking, (5) elution and USER digestion, (6) PCR amplification and deep sequencing. (**B**) Normalized 2cMeDIP-seq data generated with distinct numbers of FGSCs are shown for the indicated region. The ‘bulk’ data generated with millions of cells using the MeDIP-seq protocol is shown for comparison. (**C**) Scatter plot comparison of two 2cMeDIP-seq biological replicate datasets generated with various numbers of cells. The *r* indicates Pearson’s correlation coefficient calculated as in Figure 2C. (**D**) Bar chart showing coverage and precision of 2cMeDIP-seq signals in the indicated numbers of cells compared with the bulk counterpart. (**E**) ROC curves for the comparison for the 2cMeDIP-seq data generated by distinct numbers of cells. Values shown are AUC. Chr, chromosome. #1 and #2, two biological replicates.

We next extended our strategy to methylome profiling of low sample input with a few modifications. Essentially, two types of chemically methylated DNA fragments were supplemented into limited numbers of cells, followed by methylated DNA immunoprecipitation and high-throughput sequencing (Figure 5A). This modified approach was accordingly referred to as 2cMeDIP-seq.

We pretested various amounts of chemically methylated DNA fragments used in the immunoprecipitation procedure and found that the addition of 50 ng methylated lambda DNA yields the most optimal enrichment (Figure S5A). Using 2cMeDIP-seq, we investigated DNA methylation profiles of various numbers (50, 200, and 1000) of FGSCs [32]. Normalized DNA methylation signals showed consistency among samples of various sizes (Figure 5B). We observed that the 2cMeDIP-seq datasets were highly correlated between biological replicates (*r* = 0.962 for 1000 cells, *r* = 0.917 for 200 cells, and *r* = 0.920 for 50 cells) (Figure 5C) and fairly correlated with the MeDIP-seq data of the bulk sample (Figure S5B). Moreover, 71–89% of DNA methylation signals detected by conventional MeDIP-seq in the bulk sample could be recaptured by 2cMeDIP-seq in samples ranging from 50 to 1000 cells (Figure 5D). Using the DNA methylation data generated with the bulk sample as standard, we performed ROC curve analysis to evaluate the quality of 2cMeDIP-seq data and found that it performed well (Figure 5E). These results suggested that 2cMeDIP-seq is suitable for methylome profiling in small numbers of cells.

### 2.6. The Unique DNA Methylation Signature during FGSC Differentiation

**Figure 6:**
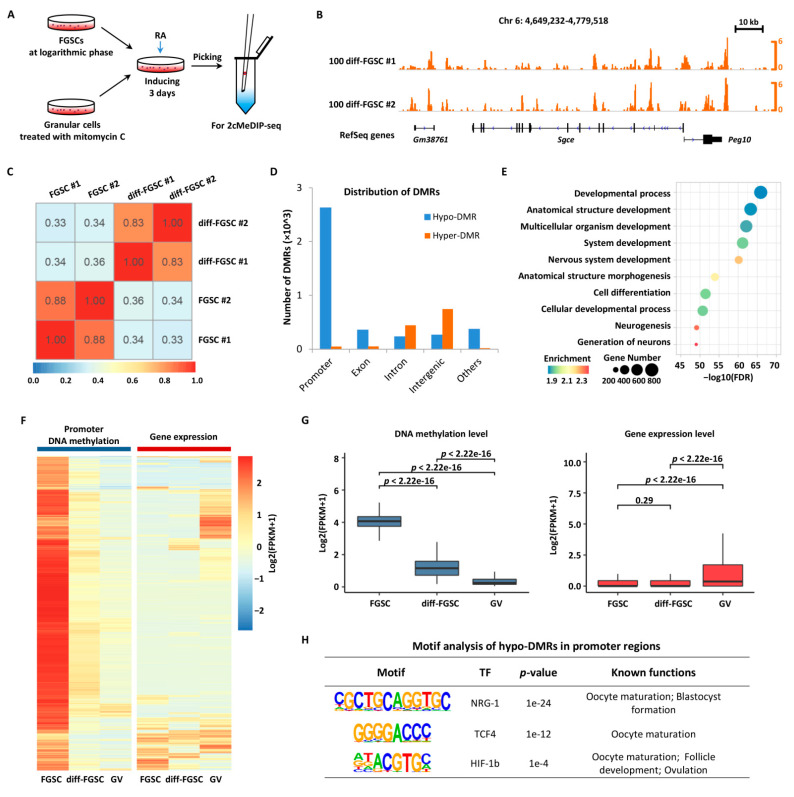
Distinct DNA methylation patterns during FGSC differentiation. (**A**) Experimental design and DNA methylation data generated with differentiated FGSCs. FGSCs were treated with RA for 3 days and collected for 2cMeDIP-seq. (**B**) Normalized 2cMeDIP-seq signals generated with 100 differentiated FGSCs are shown for the indicated region. (**C**) Heatmap showing the methylation signals correlation between the undifferentiated and the differentiated FGSCs. The correlation coefficient was calculated in non-overlapping 10 kb windows across entire genome. (**D**) The bar chart showing the numbers of DMRs distributed in distinct genomic regions of differentiated FGSCs when compared with the undifferentiated FGSCs [FDR (false discovery rate) < 0.001]. (**E**) GO analysis of genes with hypo-DMRs in promoter regions. The color and the dot size represent the enrichment levels and the number of DMR-associated genes within each GO term, respectively. (**F**) DNA methylation and expression analysis of genes with hypomethylated promoters. (**G**) Boxplots of DNA methylation or gene expression levels of the genes with hypomethylated promoters in FGSCs, diff-FGSCs, and GV oocytes. (**H**) Motifs analysis of hypomethylated DMRs in promoter regions.

DNA methylation is of particular importance in germline development [33,34]. We previously reported that DNA methylation contributes to the maintenance of mouse FGSC identity [35]. It remains unclear whether DNA methylation is involved in FGSC differentiation. To address this issue, we induced the differentiation of FGSCs in vitro (Figure 6A) with the method we recently described [36]. After three-day induction, the expression of differentiation-related genes *Stra8* and *Sycp3* was detected in retinoic acid (RA)-treated FGSCs (Figure S5C,D). We manually collected 100 differentiated FGSCs and performed methylome profiling analysis with 2cMeDIP-seq (Figure 6B).

We compared the DNA methylation patterns between undifferentiated and differentiated FGSCs. Although two biological replicates of each cell population exhibited high correlation, the methylome of undifferentiated FGSCs was poorly correlated with that of the differentiated counterpart (Figure 6C), indicating that DNA methylation undergoes widespread changes during FGSC differentiation. We then identified the differentially methylated regions (DMRs) across the genome (Table S5). Compared with undifferentiated FGSCs, 3839 hypomethylated DMRs (corresponding to 3154 genes) and 1302 hypermethylated DMRs (corresponding to 1128 genes) were identified in differentiated FGSCs. We examined the genomic distribution of the identified DMRs and found 68% of the DMRs in the hypomethylated promoter regions (Figure 6D and Figure S5E). We performed a gene ontology (GO) analysis of these genes with hypomethylated DMRs in promoter regions and found that the majority of the top 10 GO terms were development-related biological processes, including cell differentiation (Figure 6E). These findings suggest that DNA demethylation is potentially involved in FGSC differentiation.

In a recent report, we demonstrated that FGSCs could be differentiated into germinal vesicle (GV) oocytes [36]. To further understand the role of DNA methylation dynamics during FGSC development, we compared DNA methylation patterns of hypomethylated promoters in differentiated FGSCs with their counterparts in FGSCs and GV oocytes, and the expression of the corresponding genes was also analyzed (Figure 6F). Among 2627 methylated promoter regions (corresponding to 2415 genes) in FGSCs, while hypomethylated in differentiated FGSCs, most of these demethylated genes remained transcriptionally unchanged upon FGSC differentiation; instead, some of them were significantly upregulated in GV oocytes (*p* < 2.22 × 10^−16^) (Figure 6G). Considering that genomic regions with low DNA methylation level have been recognized as predictors of transcription factor binding sites [37], we examined motifs of transcription factors in these hypo-DMRs in promoter regions. We found that the motifs of the transcription factors involved in meiosis and oocyte maturation (including NRG1 [38], TCF4 [39], and HIF-1b [40]) were significantly more abundant (Figure 6H).

## 3. Discussion

In this study, we presented a 2cChIP-seq approach for generating high-quality epigenomic profiles of histone modifications with high sensitivity and robustness. Unlike the recently reported ChIL-seq in which antibody-conjugating ChIL probe–transposase complex may preferentially target nucleosome-depleted regions due to accompanying dissociations from the target chromatin [16], the 2cChIP-seq technique developed in this study is based on immunoprecipitation and more faithful in the preservation of the epigenomic features (Figure 3A,B).

So far, ‘carrier strategy’ has been frequently used for low-input ChIP-seq methods. For instance, the addition of a carrier improves immunoprecipitation efficacy and enables ChIP-seq with 500–10,000 cells [26]. Alternatively, supplementation of carrier DNA facilitates library construction in RP-ChIP-seq that may produce epigenomic profiles in 500 cells [41]. In this study, we made full use of this strategy in the epigenomic profiling of histone and DNA methylation by combining two types of carriers: (1) dUTP-containing lambda DNA that reduces sample loss during sample preparation and library construction and (2) chemically modified peptides or methylated DNA fragments which increase immunoprecipitation efficacy. Such improvements ended up with high sensitivity and fair reproducibility (Figure 2, Figure 3, Figure 5). The epigenomic signal coverage ratio generally dropped down as the number of cells decreased; we found 2cChIP-seq data derived from 1000 cells were highly representative, which could recapture more than 90% of signals identified in bulk-cell samples (Figure 2C and Figure 3A). In contrast to the reported carrier-assisted methods [11,41], in our method, the carrier materials were almost removed in the sequencing libraries (Figure 1C), which greatly reduced the sequencing costs. Thus, these improvements enable 2cChIP-seq to start with as few as 10 cells. By introducing Tn5 transposase-based cellular indexing and tagmentation, 2cChIP-seq could also be applied to single-cell level analysis (Figure 4).

The dynamics of DNA methylation are critically involved in the germ cell development [33,34]. In this study, we obtained the in vitro differentiated FGSCs as we reported previously [36] and characterized DNA methylation patterns among FGSCs, differentiated FGSCs, and GV oocytes. Compared with FGSCs, the transcription levels of demethylated genes were not obviously changed in differentiated FGSCs and became up-regulated in GV oocytes (Figure 6G). Our findings suggested that these differentiation-related genes exhibited delayed expression changes in the later development stage after methylation changes in the prior stage, a scenario observed during B-cell development [42].

DNA demethylation provides a genetic base for the binding of transcription factors, and then primes their target *cis*-regulatory elements at earlier stages during cell fate decisions [37,42]. In our study, we observed a more frequent presence of the motifs specific for the meiosis- and ovulation-related transcription factors present in hypomethylated DMRs located in promoter regions of differentiated FGSCs’ genome (Figure 6H). The potential bindings of these transcription factors are likely involved in the development of FGSCs.

## 4. Materials and Methods

Buffer solutions used in the experiment are listed in the Supplementary Materials and Methods (Supplementary Section S1.1).

### 4.1. Cell Preparation

#### 4.1.1. K562 Cell Culture

K562 cells were cultured in RPMI 1640 medium (Gibco, Carlsbad, CA, USA) containing 10% fetal bovine serum (FBS, Gibco) and 1% penicillin–streptomycin (Gibco).

#### 4.1.2. Embryonic Stem Cell Culture

E14TG2a (E14) murine ESCs were kindly provided by Prof. Jian Yang of Tongji University School of Medicine and cultured in M15 medium [43], which is described in Supplementary Section S1.2.

#### 4.1.3. Female Germline Stem Cell Culture

FGSCs were isolated as we previously reported [44] and cultured on Sandos inbred mouse (SIM) embryo-derived thioguanine- and ouabain-resistant (STO) feeder cells in a medium consisting of MEM α (Gibco), 10% FBS (Gibco), and several cell growth factors. A detailed description is given in Supplementary Section S1.3.

#### 4.1.4. Female Germline Stem Cell Differentiation

FGSCs differentiation was induced according to our previously described protocol with minor modifications [36]. The detailed procedure was conducted as described in Supplementary Section S1.4.

#### 4.1.5. Germinal Vesicle Oocyte Collection

GV oocytes collection was performed as we described previously [45,46], according the procedure described in Supplementary Section S1.5.

### 4.2. Carrier Preparation

Two commercially available histone peptides with distinct chemically modified epigenetic marks, i.e., H3K4me3 peptide (Epigentek, Farmingdale, NY, USA) and H3K27ac peptide (abcam, Cambridge, MA, USA) were used.

The dUTP-containing lambda DNA fragments with the size of 2 kb were generated by PCR amplification using primers in Table S6 and dNTP mixture (dTTP replaced by dUTP). The methylated lambda DNA (dUTP-containing) fragments were generated with CpG methyltransferase (Fermentas, Vilnius, Lithuania) and sonicated at 30% power for 10 cycles (5 s pulse and 15 s rest per cycle) with a sonicator (BRANSON, Brookfield, CT, USA) to obtain fragments with the size of 200–500 bp.

### 4.3. Low-Input 2cChIP-seq

Ten thousand K562 cells were crosslinked in 1% formaldehyde for 10 min and the reaction was terminated by supplementing 0.125 M glycine and maintaining for 10 min. After washing with PBS, the cells were centrifugated at 700× *g* for 5 min and resuspended with 500 μL low-salt lysis buffer containing protease inhibitor.

The cells mixed with 1 μg dUTP-containing lambda DNA were treated with a sonicator at 30% power for 8 cycles with a parameter of 30 s on and 59 s off per cycle to obtain genomic DNA fragments with the proper length. From this stock chromatin preparation, samples equivalent to 1000, 100, 50, and 10 cells were separated for 2cChIP-seq. Ninety percent of fragmented DNA was immunoprecipitated and the rest was kept as input.

Subsequently, 20 μL protein A + G magnetic beads (Merk Millipore, Darmstadt, Germany) were added for each ChIP assay. The beads were washed twice using 1 mL low-salt lysis buffer and resuspended with 800 μL low-salt lysis buffer, followed by adding 5 μg anti-H3K4me3 antibody (abcam) or 5 μg anti-H3K27ac antibody (abcam) and rotating for 2–4 h at 4 °C. The antibody–bead complexes were washed two times using low-salt lysis buffer, and then incubated with fragmented chromatin and the chemically modified histone peptides equaling to millions of cells (H3K4me3: 0.2 ng; H3K27ac: 0.1 ng) [26] in 800 μL low-salt lysis buffer at 4 °C with gentle rotation overnight.

The chromatin–antibody–bead complexes were washed twice separately using the following four different buffer solutions (Supplementary Section S1.1): low-salt lysis buffer, high-salt lysis buffer, LiCl buffer, and TE buffer, followed by de-crosslinking at 55 °C for 6 h in 400 μL elution buffer containing 0.25 mg/mL proteinase K. Input samples were de-crosslinked under the same conditions.

ChIP and input DNA were purified through phenol/chloroform/isoamylalcohol (25:24:1) extraction. To improve the efficiency of ethanol precipitation and the subsequent library construction process, 50 ng dUTP-containing lambda DNA was added to each sample. The purified DNA was used for library construction with NEBNext^®^ Ultra™ II DNA Library Prep Kit (New England Biolab, Ipswich, MA, USA), following the manufacturer’s instructions with modifications. Briefly, the end repair, dA-tailing, and adaptor ligation reactions were conducted according to the instruction manual, followed by purification using 0.8 × AMPure XP beads (Beckman, Brea, California, USA). The ligation products were treated with 5 μL USER enzyme (NEB) at 37 °C overnight, followed by PCR amplification with Q5^®^ High-Fidelity DNA Polymerases (NEB) for 15–20 cycles. The amplified 2cChIP-seq libraries, purified using 0.8 × Ampure XP DNA purification beads, were sequenced with Illumina HiSeq X-ten.

### 4.4. Single-Cell 2cChIP-seq

#### 4.4.1. Preparation of Barcoded Tn5 Transposome Complexes

The Tn5 transposome was assembled using TruePrep Tagment Enzyme kit (Vazyme, Nanjing, China) according to the manual with modifications. Briefly, each T5 or T7 oligo was mixed with equal mole of common annealing primer (Table S3) and incubated for 5 min at 95 °C, followed by a programmed temperature decrease of 0.1 °C/s to 25 °C. The 8.75 μM barcoded Tn5 transposome was obtained by mixing 10 μL TruePrep Tagment Enzyme (2 μg/μL) and 10 μL preannealed oligonucleotides at 35 μM with 20 μL storage buffer, and incubating at 25 °C for 1 h.

The activity of the assembled Tn5 complex was detected through mouse genomic DNA tagmentation as described previously [47]. In each reaction, 300 ng genomic DNA was mixed with various amounts of Tn5 transponsome and the result is shown in Figure S4A. We chose 1 μg Tn5 transposase (17.5 μM) for tagmentation, as this condition yielded DNA fragments of less than 1 kb.

#### 4.4.2. Single-Cell Indexing and Library Preparation

As described previously [18], 0.3% SDS was used for single-cell chromatin opening. After treatment with 10% Triton X-100 to quench SDS, each single cell was supplemented with 2 μL TMgCl-DMF, 0.875 μM Tn5-T5, and 0.875 μM Tn5-T7. The final volume of tagmentation reaction was 10 μL by supplementing with ddH_2_O, and the mixture was incubated at 37 °C for 50 min and 55 °C for 10 min. The reaction was terminated by adding 2 µL 250 mM EDTA and incubating at room temperature for 20 min.

After adding 5 ng dUTP-containing lambda DNA into each well, single cells in a 96-well plate were pooled and centrifuged in 1.5 mL tubes. The pellets were resuspended in 20 μL releasing buffer, incubated for 30 min at room temperature, and sonicated at a low level for 5 cycles (15 s pulse and 30 s pulse per cycle; Bioruptor plus, Diagenode, Liege, Belgium) to release chromatin. Then 100 μL ChIP dilution buffer was added to the cell mixture, and centrifugation at 4 °C, 20,000× *g* for 15 min was performed to collect soluble chromatin from about 100 cells, which was used as starting material for low-input 2cChIP-seq analysis.

Finally, ChIP DNA was amplified with 1 μL of 25 μM i5 index primer, 1 μL of 25 μM i7 index primer (Table S3), and 25 μL of 2 × KAPA master mix in 50 μL solution. The PCR reaction was performed at 72 °C for 5 min, 98 °C for 45 s, 20 cycles of 98 °C for 15 s, 63 °C for 30 s, 72 °C for 1 min, and a final extension at 72 °C for 5 min. The purified libraries were subjected to Illumina Nextseq 500 for sequencing.

### 4.5. Low-Input 2cMeDIP-seq

Before genomic DNA was extracted from small numbers of FGSCs (50, 200, and 1000 cells) or 100 differentiated FGSCs using GenElute™ Mammalian Genomic DNA Miniprep Kit (Sigma-Aldrich, St. Louis, MO, USA), 1 μg dUTP-containing lambda DNA was mixed into the limited numbers of cells to reduce sample loss. The DNA mixture was subsequently sheared into fragments with suitable lengths by the 40-cycle sonication at 30% power (5 s on and 15 s off per cycle; BRANSON). The sonicated genomic DNA was subjected to library preparation with NEBNext^®^ Ultra™ II DNA Library Prep Kit for Illumina (NEB), and the libraries were purified with 1 × AMPure XP beads.

The immunoprecipitation procedure was conducted according to a reported MeDIP protocol [48] with slight modifications. Briefly, methylated lambda DNA was supplemented into the library before DNA denaturation. We optimized the amount of spike-in methylated lambda DNA (dUTP-containing) by 2cMeDIP-qPCR (Table S6) and examined the enrichment effect in FGSCs. After optimization, 50 ng dUTP-containing methylated lambda DNA was mixed with adaptor-ligated DNA and denatured for 10 min at 95 °C. The mixture was then immediately incubated in an ice bath for 10 min, and 1/10 volume of the denatured product was set aside as input. The protein A + G magnetic beads (Millipore, Darmstadt, Germany) were incubated with anti-5-methylcytosine monoclonal antibody (Epigentek, Farmingdale, NY, USA) at 4 °C for 2–4 h with overhead shaking. Subsequently, the library was added to the antibody–bead complexes, and the mixture was incubated at 4 °C overnight under gentle rotation.

After immunoprecipitation, the dynabead–antibody–methylated DNA complexes were washed four times with wash buffer, followed by proteinase K treatment for 3 h at 55 °C. The immunoprecipitated DNA was extracted by phenol/chloroform/isoamylalcohol, precipitated by ethanol, and dissolved in 10 mM Tris-HCl (pH 8.0). The purified methylated DNA and input DNA were treated with USER enzyme (NEB), followed by 15–20 cycles of PCR amplification utilizing Q5 High-Fidelity DNA Polymerase (NEB). The purified 2cMeDIP-seq libraries were sequenced by Illumina HiSeq X-ten.

### 4.6. RNA Isolation and RT-PCR

Total RNA was isolated utilizing a PicoPure RNA Isolation Kit (Thermo Fisher Scientific, Waltham, MA, USA), followed by RT-PCR as we described previously [49]. The detailed procedure is shown in Supplementary Section S1.6.

### 4.7. Immunofluorescent Staining

Cells were fixed with 4% paraformaldehyde and processed as described in Supplementary Section S1.7.

### 4.8. Low-Input RNA-Seq

Basically, the RNA-seq library of 5–8 differentiated FGSCs was constructed according to Smart-seq protocol with modifications [50]. A detailed description can be found in Supplementary Section S1.8.

### 4.9. Data Analysis

#### 4.9.1. Reads Mapping

The raw sequencing reads were trimmed with trimmomatic v0.38 [51] to remove adapter sequences and low-quality reads. Then the clean reads were mapped against the reference genome (hg19 or mm10) by Bowtie v1.2.1.1 [52] with parameters ‘-l 50 -n 2 -X 600 --quiet --no-unal -m 1′. Reads mapped to the same location and orientation were removed in the downstream analysis. The presence of phage lambda DNA fragments was determined once the reads were aligned to the phage lambda DNA genome (NCBI, https://www.ncbi.nlm.nih.gov/nuccore/NC_001416.1, accessed on 28 March 2021). The bigwig files were generated with the function *bamCoverage* of software from deepTools [53] using the following parameters (--binSize 200 and --smoothLength 1000), and ‘binSize 1000′ was used for 2cMeDIP-seq data. All heatmaps of H3K4me3 and H3K27ac data were generated using deepTools. The histone marks of low input were aligned around the center (±3 kb) of the bulk samples’ peaks.

#### 4.9.2. Peak Calling

Peaks were defined using MACS v2.2.1 [54]. The default parameters were used for calling peaks in bulk-cell samples except ‘-q 0.01′. The q values for peak calling in the limited number of cells were determined by ROC curves according to a recent report (e.g., q-value was set as 0.23 for 10 cells) [16]. The broad peak calling model in MACS2 was used for 2cMeDIP-seq data with default parameters except ‘--broad-cutoff 1e-3′; and the parameter of ‘--broad-cutoff’ was set as 0.1 for 2cMeDIP-seq data of the limited numbers of cells. To examine the signal-to-noise ratio of our datasets, the fraction of reads in peaks (FRiP) of H3K4me3 and H3K27ac data was calculated using bedtools v2.25.0 [55]. The CEAS v0.9.9.7 [56] was implemented to annotate the genomic distribution of peaks in H3K4me3 and H3K27ac datasets.

#### 4.9.3. Correlation Analysis and Construction of Receiver Operating Characteristic Curves

To evaluate the robustness and reliability of our method, we calculated the correlation of either histone modifications or DNA methylation data, respectively. Pearson’s correlation coefficients were analyzed in 4-kb bins across the entire human or mouse reference genome.

The performance of our method was compared with the other two state-of-the-art methods, ChIL-seq [16] and uliCUT&RUN [23], using ROC curves. We focused on the promoter region (from 2000 bp upstream to 500 bp downstream of a transcription start site). The ENCODE ChIP-seq data (GSM2534289 for H3K4me3; GSM733656 for H3K27ac) or bulk MeDIP-seq data were used as the gold standard, respectively. The gold-standard true positives were designated as the identified promoter peaks present in gold-standard samples. The identified promoter peaks that did not overlap with the peaks in bulk samples were regarded as gold-standard negative sets. We generated the ROC curves for each method by calculating the true positive and false positive rates with different q-value cutoffs [10].

#### 4.9.4. Single Cell 2cChIP-seq Data Analysis

##### Reads Mapping of Single-Cell 2cChIP-seq Data

The raw sequencing reads were trimmed using cutadapt v2.10 [57] to remove adapter and low-quality reads. Then the qualified reads were mapped against the mm10 reference genome by Bowtie2 v2.3.3.1 [58] with default parameters. Reads mapped to the same location and orientation were removed in the downstream analysis.

##### Sensitivity and Precision Analysis of Single-Cell 2cChIP

The sensitivity and precision of single-cell 2cChIP-seq data were calculated as described previously [11,24]. Briefly, the ratio of the reference peak regions recovered by single-cell reads was used to evaluate the sensitivity, and the ratio of single-cell reads located in the reference peak regions was used to verify the precision. Among the sensitivity and precision data of all single cells, those for the top 5% of single cells were plotted in R (version 3.6.3). We used bulk ESC H3K4me3 ChIP-seq data from ENCODE (GSM1003756) as a reference and obtained a simulated random profile as the control.

##### Correlation Analysis of Single-Cell 2cChIP-seq Data

A genome-wide correlation was conducted to assess the distribution of reads across samples. Pearson correlation coefficients between two independent experiments of single-cell 2cChIP-seq data or between bulk ChIP-seq data and pooled single-cell 2cChIP-seq data were calculated.

##### Plotting Transcription Start Site Profiles of Single-Cell 2cChIP-seq Data

For each library, the average TSS density was analyzed using deepTools. In particular, the region of 3 kb around each TSS was taken into account and divided into 200 bp bins. According to the data analysis method of scChIC-seq [24], the density profile was obtained by dividing the number of reads mapped to the bin by the total number of mapped reads, and averaging over all promoters.

#### 4.9.5. Identification of Differentially Methylated Regions

DNA methylation peaks were called with MACS2 and the DMRs were identified with DiffBind [59] and DESeq2 packages [60]. Only peaks with more than 10 counts were chosen for DMR identification using the DESeq2 package. Lastly, we classified the identified DMRs (|log2 FC (fold change)| ≥ 1) into the following groups: promoter (from 2000 bp upstream to 500 bp downstream of a TSS), exonic, intronic, and intergenic regions of genes, using the script annotatePeaks.pl in HOMER toolkit.

#### 4.9.6. Gene Ontology Enrichment Analysis

To understand the biological relevance of DNA methylation dynamics to female germ cell development, we performed GO enrichment analysis of methylated genes at different developmental stages of germ cells. The genes with promoter containing hypo-DMRs were subjected to GO enrichment analysis with the gene ontology resource [61].

#### 4.9.7. Low-Input RNA-Seq Read Alignment and Quantification

Alignment of the reads and calculation of gene expression were performed with the Tuxedo pipeline (Tophat, Cufflinks) [62]. Raw sequencing reads were aligned to the mm10 reference genome with TopHat v2.1.1. Gene expression levels were quantified from the mapped reads with Cufflinks v2.2.1 and expressed as fragments per kilobase exon model per million mapped reads (FPKM). Cuffdiff in Cufflinks was performed for differential expression analysis. Genes with *p*-value < 0.01 and fold change > 2 were thought to be expressed differentially.

#### 4.9.8. Motif Enrichment

The promoter regions with hypo-DMRs during FGSC differentiation were used for transcription factor motif analysis. Enrichment for TF motifs was carried out through findMotifsGenome.pl in Homer v4.11 [63] with parameter ‘-size given’.

#### 4.9.9. Data Access

The previously published sequencing datasets used in this study are available under accession numbers GSM2534289 (ENCODE ChIP-seq; K562 cells; H3K4me3), GSM733656 (ENCODE ChIP-seq; K562 cells; H3K27ac), GSE115047 (ChIL-seq; C2C12 cells; H3K4me3 and H3K27ac for 100 and 1000 cells) [16], GSE111121 (uliCUT&RUN; ESC; H3K4me3 for 10 and 50 cells) [23], GSM1003756 (ENCODE ChIP-seq; ES cells; H3K4me3), SRP066132 (RNA-seq; FGSCs) [35], and GSE75738 (RNA-seq; GV cells) [45].

## 5. Conclusions

This study provided a technique that allows the generation of high-quality epigenomic profiles of histone modifications or DNA methylation from low-input samples and histone modification analysis from single cells, and thus paved the way for elucidating chromatin state in low numbers of cells.

## Data Availability

All original sequencing data generated in this study are available in the Gene Expression Omnibus database under accession number GSE185619, (https://www.ncbi.nlm.nih.gov/geo/query/acc.cgi?acc=GSE185619, accessed on 16 November 2021) with the token of ‘edinoqwirpehjkf’. The code for sequencing data analysis can be found at https://github.com/Junwu302/DataAnalysisFor2cChIP (accessed on 2 April 2021).

## Supplementary Materials

The supporting information can be downloaded at: https://www.mdpi.com/article/10.3390/ijms232213984/s1.
