# Supplementary material for: 2cChIP-seq and 2cMeDIP-seq: The Carrier-Assisted Methods for Epigenomic Profiling of Small Cell Numbers or Single Cells
Source: Int J Mol Sci. 2022 Nov 12;23(22):13984. doi: 10.3390/ijms232213984 (PMC9692998; doi:10.3390/ijms232213984)
Supplement: Supplementary file 1 [file ijms-23-13984-s001.zip › ijms-1946430-supplementary.pdf]

S1. Supplemental Materials and Methods

S1.1 Buffer solutions used in the experiment

Low-salt lysis buffer: 50 mM HEPES KOH pH 7.5, 150 mM NaCl, 1 mM EDTA, 1% Triton X-100, 0.1% SDS, and 0.1% sodium deoxycholate;

High-salt lysis buffer: 50 mM HEPES KOH pH 7.5, 350 mM NaCl, 1 mM EDTA, 1% Triton X-100, 0.1% SDS, and 0.1% sodium deoxycholate;

LiCl buffer: 10 mM Tris-HCl pH 8.0, 250 mM LiCl, 1% NP40, 1% sodium deoxycholate, and 1 mM EDTA;

TE buffer: 10 mM Tris-HCl pH 8.0, and 1 mM EDTA;

Elution buffer: 25 mM Tris-HCl pH 8.0, 5 mM EDTA, and 0.5% SDS;

Storage buffer: 50 mM Hepes pH 7.2, 100 mM NaCl, 0.1 mM EDTA, 1 mM DTT, 0.1% Triton X-100, and 60% glycerol;

5 × TMgCl-DMF: 50 mM TAPS-NaOH pH 8.3, 25 mM MgCl2, and 50% DMF;

Releasing buffer: 20 mM Tris-HCl pH 8.0, 200 mM NaCl, 2 mM EDTA, and 0.8% SDS;

ChIP dilution buffer: 20 mM Tris-HCl pH 8.0, 0.01% SDS, 1% Triton X-100, 2 mM EDTA, and 200 mM NaCl;

Wash buffer: 10 mM sodium phosphate pH 7.0, 140 mM NaCl, and 0.05% Triton X-100.

S1.2 Embryonic stem cell (ESC) culture

E14TG2a (E14) murine ESCs were cultured on 0.2% gelatin-coated plates in M15 medium containing KnockOut DMEM (Gibco), 15% fetal bovine serum (FBS, Gibco), 1% non-essential amino acids (NEAA, Gibco), 1% glutamine-penicillin-streptomycin (Gibco), 0.1 mM 2-mercaptoethanol (Sigma), and 1,000 U/mL human leukaemia inhibitory factor (LIF, Millipore).

S1.3 Female germline stem cell (FGSC) culture

FGSCs were cultured on mitomycin C-treated (10 μg/mL, Sigma) mitotically inactivated STO feeder cells in the medium consisted of Minimum Essential Medium α (MEM-α, Gibco), 10% fetal bovine serum (FBS, Gibco), 1% non-essential amino acids (NEAA, Gibco), 1 mM sodium pyruvate (Sigma), 2 mM L-glutamine (L-Glu, Sigma), 0.1 mM 2-mercaptoethanol (Sigma), 10 ng/mL mouse epidermal growth factor (EGF, PeproTech), 10 ng/mL mouse leukaemia inhibitory factor (LIF, Millipore), 10 ng/mL mouse glial cell

line-derived neurotrophic factor (GDNF, PeproTech), 20 ng/mL human basic fibroblast growth factor (BFGF, PeproTech), and 1% glutamine-penicillin-streptomycin (Gibco).

S1.4 FGSC differentiation

In order to induce differentiation, FGSCs in logarithmic phase were digested with 0.5% trypsin and suspended in differentiation medium (FGSC culture medium with all supplements except GDNF, EGF, and LIF, but including 10 ng/mL bone morphogenetic protein 4 (BMP4, PeproTech) and 10 μM retinoic acid (RA, Sigma)). Single cells of FGSCs were cultured on monolayer granulosa cells at 37°C in a 5% CO_2 incubator for 3 days. We manually collected 100 differentiated cells with a diameter of about 20 μm for DNA methylation profiling analysis with 2cMeDIP-seq.

S1.5 Germinal vesicle (GV) oocyte collection

To collect GV oocytes, 6-week-old C57BL/6 female mice were injected with 8 U pregnant mare serum gonadotropin (PMSG). After 46-48 h, GV oocytes enclosed by cumulus cells were obtained by puncturing the antral follicles with 30-gauge needles. The denuded oocytes were released after adherent cumulus cells being removed by hyaluronidase treatment, and then stored at −80°C before 2cMeDIP-seq experiments.

S1.6 RNA isolation and RT-PCR

Total RNA was prepared using a PicoPure RNA Isolation Kit (Thermo Fisher Scientific). Reverse transcription was performed using a HiScript® II Q RT SuperMix kit (Vazyme), according to the manufacturer’s instructions. For RT-PCR, 35 cycles were performed using Taq polymerase (Takara) with primers for genes of interest (Table S6). The gene encoding glyceraldehyde-3-phosphate dehydrogenase (GAPDH) was used as control.

S1.7 Immunofluorescent staining

Cells were washed with PBS, fixed with 4% paraformaldehyde for 20 min and penetrated with 1% Triton X-100 for 10 min. These cells were then blocked with 5% goat serum at 37°C for 30 min, followed by incubating with the diluted (1:100) rabbit anti-Stra8 antibody (Origene) at 4°C overnight. The TRITC-labeled goat anti-rabbit antibody (1:15; Proteintech) was used for immunostaining. Images were obtained with Leica DM2500 microscope and Leica DFC 550 digital camera.

S1.8 Low-input RNA-seq

The RNA-seq libraries of 5-8 differentiated FGSCs were constructed according to Smart-seq protocol

with modifications. The dUTP-containing DNA was added to cDNA to make the amount of nucleic acid up to 100 ng. The mixture was sheared using Covaris M220, with the parameters of length 350 bp and volume 50 µL. Fragmented DNA was ligated with adaptors with NEBNext® Ultra™ II DNA Library Prep Kit (NEB) following the manufacturer's instructions. The ligation products were treated with USER enzyme (NEB) before PCR amplification. The purified RNA-seq libraries were sequenced by Illumina HiSeq X-ten platform.

S2. Supplementary Figures

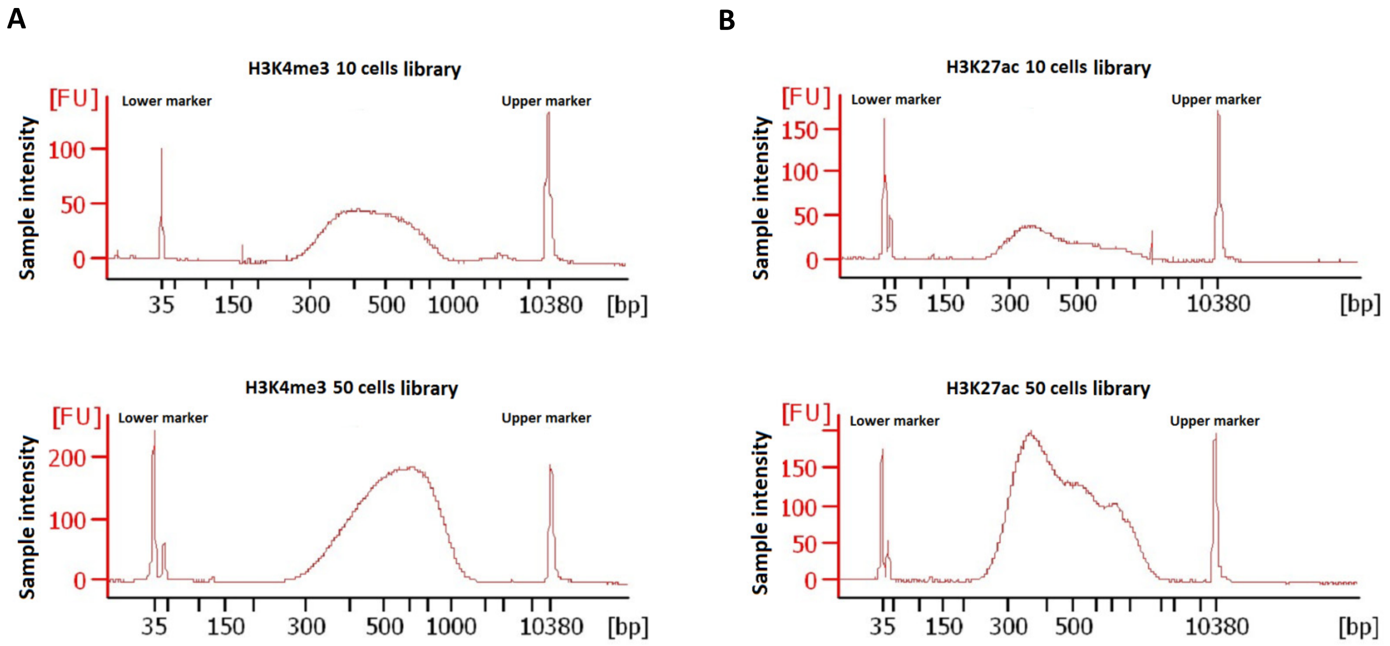

Figure S1. Distribution of the final sequencing libraries

(A) and **(B)** 2cChIP-seq libraries were evaluated by an Agilent 2100 Bioanalyzer (Agilent Technologies, CA, USA). **(A)** DNA size distribution of 2cChIP-seq H3K4me3 libraries using 10 and 50 cells. **(B)** DNA size distribution of 2cChIP-seq H3K27ac libraries using 10 and 50 cells.

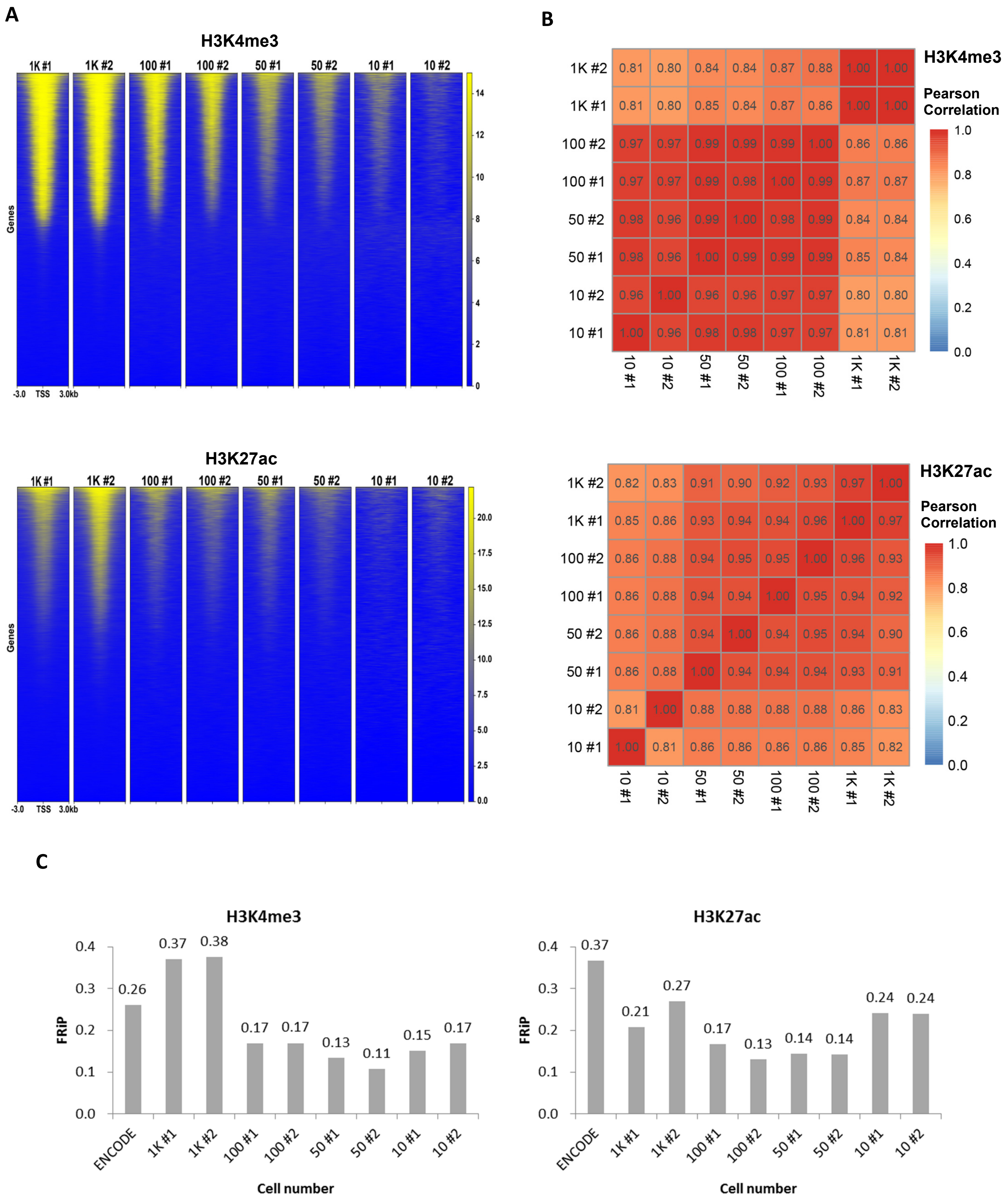

Figure S2. 2cChIP-seq generated high-quality data for histone marks in limited numbers of cells

(A) Heatmaps showing H3K4me3 and H3K27ac 2cChIP-seq signals at gene TSS ± 3 kb regions with indicated cell numbers of K562 cells. **(B)** Genome-wide correlations calculated in non-overlapped 4-kb bins between 2cChIP-seq data sets of distinct cell numbers (above: H3K4me3; below: H3K27ac). **(C)** The

fraction of reads in peaks (FRiP) of 2cChIP-seq and ENCODE data sets (H3K4me3, GSM2534289; H3K27ac, GSM733656). #1 and #2 represent two independent 2cChIP-seq experiments.

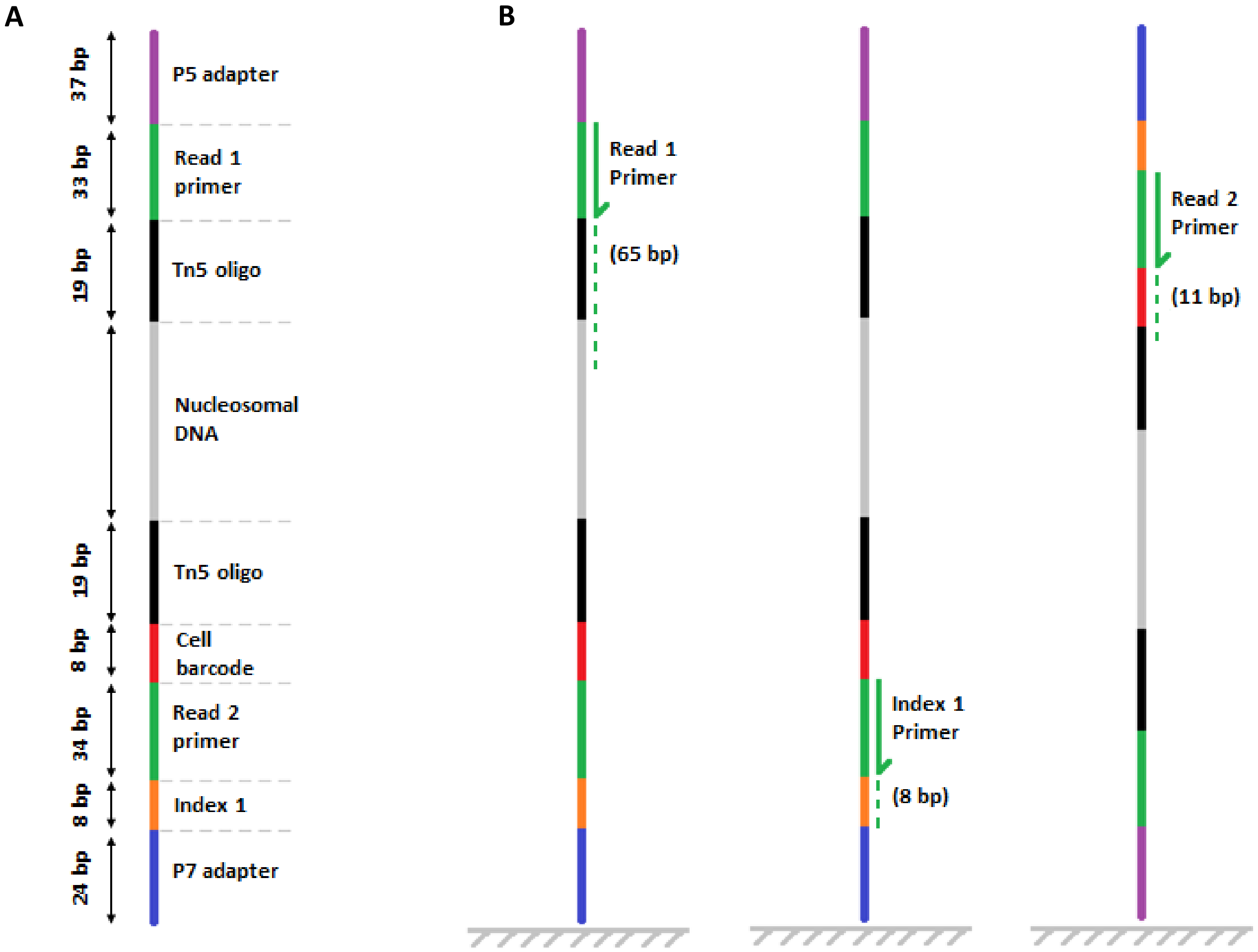

Figure S3. The structure of single-cell 2cChIP-seq library

(A) Schematic of the final sequencing product with size in bp of each constituting element. **(B)** Single-cell 2cChIP-seq libraries were sequenced as follows: 65 bp were assigned to read the nucleosomal sequence and 11 bp were assigned to read the barcode.

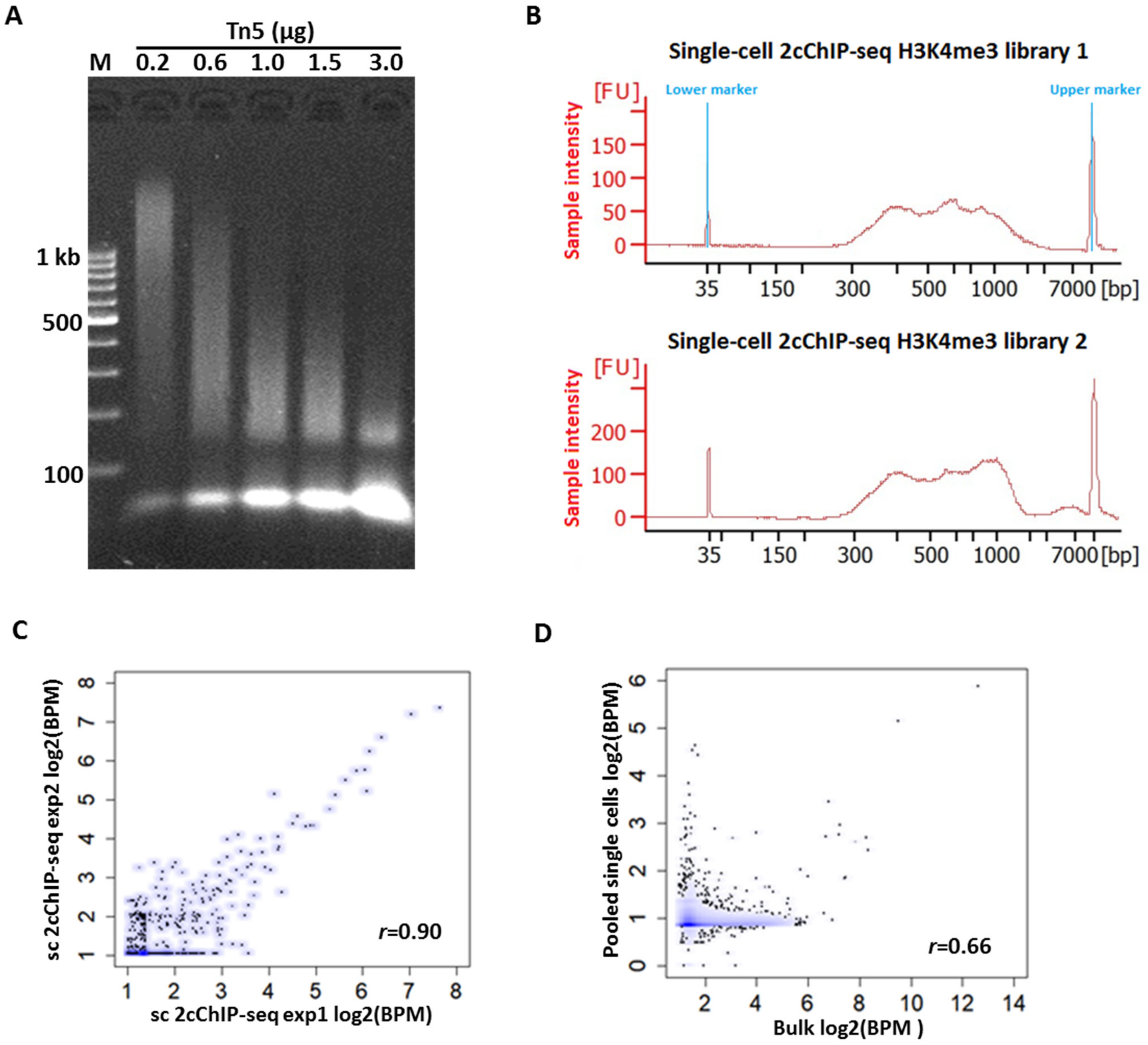

Figure S4. Single-cell 2cChIP-seq library preparation and data analysis

(A) Optimization of the Tn5 complex amount for efficient tagmentation. The condition yielding DNA fragments of < 1 kb was picked for single-cell 2cChIP-seq. 1 μg Tn5 transposase was selected accordingly; M: 100 bp DNA marker. **(B)** Single-cell 2cChIP-seq libraries were analyzed using a bioanalyzer (Agilent Technologies, CA, USA). **(C)** Comparison of single-cell 2cChIP-seq data sets from two experiments using H3K4me3 signals of aggregated single mouse embryonic stem cells in 96-well plates. Pearson correlation coefficient was calculated with H3K4me3 signals. **(D)** A scatter plot showing the correlation between the bulk cell H3K4me3 ChIP-seq (GSM1003756) and pooled single cell 2cChIP-seq data. BPM, bins per million mapped reads.

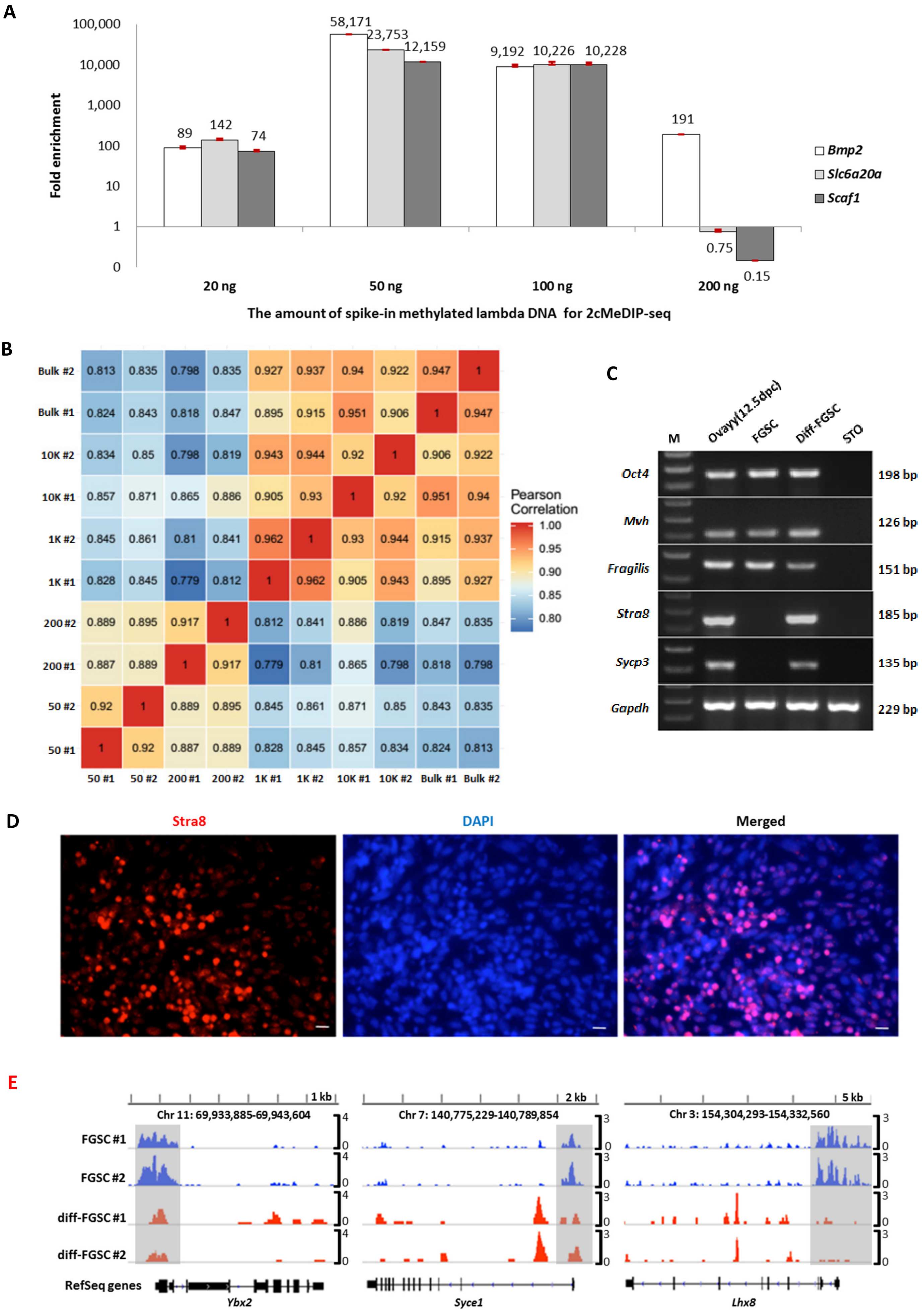
A
100,000
10,000
1,000
100
10
1
0
Fold enrichment
89
142
74
58,171
23,753
12,159
9,192
10,226
10,228
191
0.75
0.15
20 ng
50 ng
100 ng
200 ng
The amount of spike-in methylated lambda DNA for 2cMeDIP-seq
Bmp2
Slc6a20a
Scaf1
B
Bulk #2 0.813 0.835 0.798 0.835 0.927 0.937 0.94 0.922 0.947 1
Bulk #1 0.824 0.843 0.818 0.847 0.895 0.915 0.951 0.906 1 0.947
10K #2 0.834 0.85 0.798 0.819 0.943 0.944 0.92 1 0.906 0.922
10K #1 0.857 0.871 0.865 0.886 0.905 0.93 1 0.92 0.951 0.94
1K #2 0.845 0.861 0.81 0.841 0.962 1 0.93 0.944 0.915 0.937
1K #1 0.828 0.845 0.779 0.812 1 0.962 0.905 0.943 0.895 0.927
200 #2 0.889 0.895 0.917 1 0.812 0.841 0.886 0.819 0.847 0.835
200 #1 0.887 0.889 1 0.917 0.779 0.81 0.865 0.798 0.818 0.798
50 #2 0.92 1 0.889 0.895 0.845 0.861 0.871 0.85 0.843 0.835
50 #1 1 0.92 0.887 0.889 0.828 0.845 0.857 0.834 0.824 0.813
50 #1 50 #2 200 #1 200 #2 1K #1 1K #2 10K #1 10K #2 Bulk #1 Bulk #2
Pearson Correlation
1.00
0.95
0.90
0.85
0.80
C
M
Ovayy(12.5dpc)
FGSC
Diff-FGSC
STO
Oct4
Mvh
Fragilis
Stra8
Sycp3
Gapdh
198 bp
126 bp
151 bp
185 bp
135 bp
229 bp
D
Stra8
DAPI
Merged
E
1 kb
Chr 11: 69,933,885-69,943,604
2 kb
Chr 7: 140,775,229-140,789,854
5 kb
Chr 3: 154,304,293-154,332,560
FGSC #1
FGSC #2
diff-FGSC #1
diff-FGSC #2
RefSeq genes
Ybx2
Syce1
Lhx8

Figure S5. 2cMeDIP-seq generated high-quality data for DNA methylation in limited numbers of cells

(A) Optimizing the amount of methylated lambda DNA for immunoprecipitation. **(B)** Genome-wide Pearson correlation analysis (window size = 4 kb) of normalized read counts of 2cMeDIP-seq data sets in distinct sample sizes. **(C)** Detection of mRNA expression of germ cell markers and differentiation markers by RT-PCR. M: 100 bp DNA marker; lane 1, Ovary (12.5 dpc); lane 2, FGSC; lane 3, Diff-FGSC (differentiated FGSC); lane 4, STO (negative control). The sizes of the resolved DNA fragments are (in bp): *Oct4*, 198; *Mvh*, 126; *Fragilis*, 151; *Stra8*, 185; *Sycp3*, 135; *Gapdh*, 229 (internal control). **(D)** Immunofluorescence detection of Stra8 in differentiated FGSCs. Scale bars, 20 μm. **(E)** Examples of track view showing DNA methylation signals at three differentiation-related loci *Ybx2*, *Syce1*, and *Lhx8*. Gray rectangles outline the promoter regions. Chr, chromosome. #1 and #2, two biological replicates.
